# Exploratory Study of a CT Radiomics Model for the Classification of Small Cell Lung Cancer and Non-small-Cell Lung Cancer

**DOI:** 10.3389/fonc.2020.01268

**Published:** 2020-09-04

**Authors:** Shihe Liu, Shunli Liu, Chuanyu Zhang, Hualong Yu, Xuejun Liu, Yabin Hu, Wenjian Xu, Xiaoyan Tang, Qing Fu

**Affiliations:** ^1^Department of Radiology, The Affiliated Hospital of Qingdao University, Qingdao, China; ^2^Department of Ultrasound, The Affiliated Hospital of Qingdao University, Qingdao, China

**Keywords:** quantitative imaging, lung cancer histology, tomography, X ray, radiomics, classification

## Abstract

**Background:** Radiomics can quantify tumor phenotypic characteristics non-invasively by applying feature algorithms to medical imaging data. In this study, we investigated the association between radiomics features and the tumor histological subtypes, and we aimed to establish a nomogram for the classification of small cell lung cancer (SCLC) and non-small-cell lung cancer (NSCLC).

**Methods:** This was a retrospective single center study. In total, 468 cases including 202 patients with SCLC and 266 patients with NSCLC were enrolled in our study, and were randomly divided into a training set (*n* = 327) and a validation set (*n* = 141) in a 7:3 ratio. The clinical data of the patients, including age, sex, smoking history, tumor maximum diameter, clinical stage, and serum tumor markers, were collected. All patients underwent enhanced computed tomography (CT) scans, and all lesions were pathologically confirmed. A radiomics signature was generated from the training set using the least absolute shrinkage and selection operator algorithm. Independent risk factors were identified by multivariate logistic regression analysis, and a radiomics nomogram based on the radiomics signature and clinical features was constructed. The capability of the nomogram was evaluated in the training set and validated in the validation set.

**Results:** Fourteen of 396 radiomics parameters were screened as important factors for establishing the radiomics model. The radiomics signature performed well in differentiating SCLC and NSCLC, with an area under the curve (AUC) of 0.86 (95% CI: 0.82–0.90) in the training set and 0.82 (95% CI: 0.75–0.89) in the validation set. The radiomics nomogram had better predictive performance [AUC = 0.94 (95% CI: 0.90–0.98) in the validation set] than the clinical model [AUC = 0.86 (95% CI: 0.80–0.93)] and the radiomics signature [AUC = 0.82 (95% CI: 0.75–0.89)], and the accuracy was 86.2% (95% CI: 0.79–0.92) in the validation set.

**Conclusion:** The enhanced CT radiomics signature performed well in the classification of SCLC and NSCLC. The nomogram based on the radiomics signature and clinical factors has better diagnostic performance for the classification of SCLC and NSCLC than the simple application of the radiomics signature.

## Introduction

Lung cancer is the most common malignant tumor in the world, ranking first in cancer-related deaths (1, 2). One study showed that the annual survival rate of lung cancer patients after early diagnosis and treatment can be increased from 14 to 49% (3). There are two main types of lung cancer: small cell lung cancer (SCLC) and non-small-cell lung cancer (NSCLC) (4). SCLC is highly malignant and sensitive to radiotherapy and chemotherapy (5); NSCLC is relatively less malignant, and the probability of early metastasis is relatively low. It is not as sensitive to chemoradiotherapy as SCLC (6). Treatment for SCLC is mainly based on chemotherapy and radiotherapy (5), whereas treatment for NSCLC is mainly based on surgical resection or surgery plus radiotherapy and chemotherapy (5, 7, 8). Histological classification can help doctors determine the best treatment plan and strategy for lung cancer patients (9, 10). Currently, the most widely used methods to obtain pathological tissue are tracheoscopy and computed tomography (CT)-guided percutaneous lung biopsy (11–14). However, both of these technologies are invasive, with certain risks and high costs (15, 16). In addition, for a certain proportion of lung cancer cases adjacent to the mediastinum, aorta, and other large blood vessels, CT-guided biopsy is highly risky and difficult (16), while bronchoscopy has a low success rate in the extraction of lesions below grade 5 of the bronchus (17). Therefore, thoracic surgeons and pulmonary oncologists hope to find a non-invasive and cost-effective alternative. In recent years, a large number of basic studies have suggested that radiomics provides promising opportunities in this regard. It assesses the tumor tissue characteristics non-invasively. Furthermore, radiomics is relatively cost-effective and has been used for oncological diagnosis, staging, and treatment guidance with high accuracy (18–22).

A limited number of studies have investigated the association of radiomic features and NSCLC tumor histology (23–28). It is believed that imaging features can independently predict the histological subtypes of lesions and provide a basis for the formulation and modification of clinical treatment plans. However, because no clinical parameters were added, the prediction efficiency of these models was still not as expected (23–28). Therefore, this study aimed to establish a prediction model based on enhanced CT images and clinical features for the histological classification of SCLC and NSCLC and to preliminarily explore the clinical application value of this model.

## Materials and Methods

### Data Cohort

The protocol was approved by the Institutional Review Board of the Affiliated Hospital of Qingdao University. The need for informed consent was waived by the Institutional Review Board. A cohort of consecutive 3,971 patients with lung cancer who were confirmed by biopsy or surgery between January 2014 and June 2018 was identified for this retrospective study.

The inclusion criteria were as follows: (1) pathological confirmation of lung cancers based on the histological examination of surgical resection or biopsy specimens; and (2) availability of dual-phase contrast-enhanced CT before treatment.

The exclusion criteria were as follows: (1) no enhanced CT examination in our hospital (*n* = 1,537); (2) no thin-layer recombination images or poor image quality (*n* = 528); (3) patients with incomplete clinical data (*n* = 864); (4) patients who received previous treatment (e.g., radiotherapy, chemotherapy) before surgery (*n* = 423); (5) difficulty in precisely drawing the regions of interest (ROIs) due to small size (long diameter < 1 cm) (*n* = 166); and (6) patients with a history of other primary malignancies (*n* = 85).

**Figure 1:**
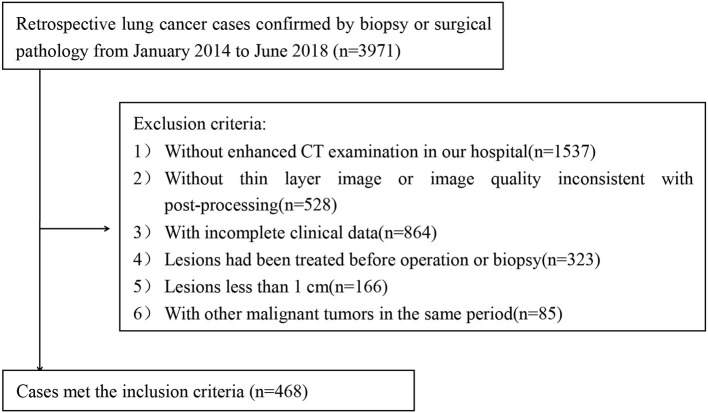
Flowchart of the study group inclusion process.

Finally, a total of 468 cases (202 patients with SCLC and 266 patients with NSCLC) were enrolled in our study (Figure 1).

The clinical data included age, sex, smoking history, clinical stage, maximum tumor diameter, and serum tumor markers [serum gastrin-releasing peptide precursor (ProGRP), squamous cell carcinoma antigen (SCCA), carcinoembryonic antigen (CEA), neuron specific enolase (NSE), and cytokeratin 19 fragment (cYFRA21-1)]. According to previous studies (29, 30), the correlation between a small amount of smoking or occasional smoking and lung cancer remains uncertain, therefore, the smoking history in this study was defined as those who had a history of smoking for more than 1 year and smoked more than 20 cigarettes per day on average based on the WHO definition of heavy smokers.

### CT Image Acquisition

**Figure 2:**
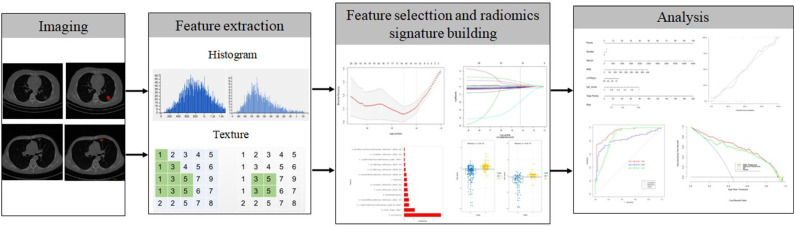
Flow chart of radiomics implementation in this study.

The radiomics workflow is displayed in Figure 2. Contrast-enhanced CT images were acquired at our hospital using either a SOMATOM (Siemens Medical Systems, Germany) scanner or a Brilliance iCT 256 (Philips Healthcare, Netherlands) scanner. The CT scanning project in our hospital was based on our country's conventional technical specifications for chest-enhanced CT scans. The scanning parameters used in this study were as follows: tube voltage, 120 kVp; detector collimation, 64 × 0.6 and 128 × 0.625 mm; pixel size, 512 × 512; slice interval, 0 mm; slice thickness, 5 mm; and reconstructed section thickness, 1 mm. Contrast-enhanced CT images were acquired after the injection of 1.0 mL/kg contrast material (iohexol injection, 300 mg/mL, Beilu Pharmaceutical Co., Ltd., Beijing, China) into the antecubital vein at a rate of 3.0–3.5 mL/s using a power injector (Ulrich CT Plus 150, Ulrich Medical), followed by a saline flush (20 mL). All patients in our cohort were scanned 25 and 70 s after injection of the contrast agent to obtain the images in the arterial phase and venous phase, respectively.

### Pathological Evaluation

According to the World Health Organization (WHO) classification of lung tumors (2015 version), all histopathological sections were retrospectively analyzed by two pathologists (WHW and JGW, with 13 and 11 years of experience, respectively, in pathological diagnosis of lung cancer). In cases of disagreement, the third pathologist (ZMW, with 19 years of experience in pathological diagnosis of lung cancer) made the final decision. All pathologists were blinded to the clinicopathological information.

### CT Radiomics Feature Extraction

Lesion outlining on CT images was performed using ITK-SNAP software (http://www.itksnap.org, version: 3.8.0, USA). The arterial and venous images were analyzed following the same procedure. One radiologist (YBH) with 8 years of experience in lung imaging interpreted CT images and outlined the edge of the target lesion. One week later, another radiologist (HLY) with 11 years of experience in lung imaging performed ROI segmentation and feature extraction independently. The two radiologists were blinded to the clinicopathological information. The lung cancer lesions were manually identified by a radiologist and confirmed by another radiologist, who were both blinded to the clinicopathological information of the patients. Each ROI was manually outlined along the margin of the lesion on the largest slice. The original images were normalized before feature extraction. Commercial software (Analysis Kit 1.0.3; GE Healthcare, China) was used to extract features. A total of 396 quantified features were extracted automatically from the delineated ROIs with four categories of radiomics features, including 10 Haralick features, 42 histograms, 9 form factors, 11 gray-level size zone matrix (GLSZM) features, 60 gray-level run-length matrix (GLRLM) features with an offset of 1/4/7, and 48 gray-level cooccurrence matrix (GLCM) features with an offset of 1/4/7.

### Development of the Radiomics Signature and Radiomics Nomogram

To reduce overfitting and select the most informative clinical and radiomics features to develop a predictive model, the least absolute shrinkage and selection operator (lasso) regression method was utilized to select the most valuable features from the primary datasets. These radiomics features with non-zero coefficients were thus selected, and radiomics scores (Rad-scores) were calculated for each patient using a linear combination of the selected features that were weighted by their respective coefficients. The diagnostic performance of the radiomics signature was quantified by the area under the receiver operating characteristic (ROC) curve (AUC) in the primary cohort and then validated in the validation cohort.

For validation, we evaluated the Rad-score difference between the two classes and used the “compare the mean between two groups” method to calculate the sample size of the validation cohorts, which satisfied the statistical power of more than 0.8. In our study, the difference in Rad-score between the two groups was 1.5. The necessary sample size of the validation cohort was 44 and we used 141 cases to validate the model. We did not retrain the model in the validation cohort. We used the cutoff obtained from the training cohort to calculate the metrics in the validation cohort.

Clinical risk factors for SCLC, including sex, age, tumor maximum diameter, smoking, clinical stage and tumor marker indicators, were first assessed in the primary cohort by using correlation analysis and multiple logistic regression analysis. Clinical features with *P* < 0.05 and the radiomics signature were applied to develop a diagnostic model for distinguishing SCLC and NSCLC by using multivariate logistic regression in the primary cohort. Backward stepwise selection was applied using a likelihood ratio test with Akaike's information criterion as the stopping rule.

To provide clinicians with a quantitative tool to predict the pathological type of lung cancer, a radiomics nomogram was built on the basis of the multivariable logistic analysis in the primary cohort. Rad-scores were also calculated in the validation set by using the algorithm built with the training set.

### Validation and Assessment of the Radiomics Nomogram

The diagnostic value of the radiomics nomogram was assessed in both the training and validation cohorts regarding discrimination, calibration and clinical value. The discrimination performance of the radiomics nomogram was quantified using ROC curves and AUC values. Calibration curves were plotted to evaluate the goodness-of-fit of the radiomics nomogram, and the Hosmer-Lemeshow test was also performed (a non-significant test statistic implies that the model calibrates well). To estimate whether the nomogram is sufficiently robust for clinical use, decision curve analysis (DCA) was applied to calculate the net benefits for a range of threshold probabilities in both the training and validation sets. The net benefit was assessed by calculating the difference between the true-positive rate and weighted false-positive rate across different threshold probabilities in the validation set.

### Statistical Analysis

The differences in continuous variables were analyzed by an independent *t*-test. Fisher's exact test or the chi-square test was used for categorical variables. The diagnostic performance of the multivariate models was evaluated using ROC analysis and AUC values. The diagnostic sensitivity, specificity, accuracy, positive likelihood ratio, and negative likelihood ratio were also calculated.

The intraclass correlation coefficient (ICC) was calculated to evaluate the interobserver variability of radiomics feature extraction. Radiomics features with ICC values no lower than 0.75 were regarded as highly reproducible features.

All statistical analyses were performed using R statistical software (http://www.Rproject.org, version 3.4.4). Lasso regression was performed using the “glmnet” package. Multivariate logistic regression, nomogram construction, and calibration plot construction were performed using the “rms” package. DCA was performed using the “dca.r” function. ROC curves were drawn and analyzed using the “proc” package. A two-tailed *P* < 0.05 was considered statistically significant.

## Results

### Comparison of Clinical Factors Between SCLC and NSCLC Patients

The results showed that there was a statistically significant difference in the proportion of smoking between SCLC and NSCLC patients (*P* < 0.001), and there was no statistically significant difference in sex, age, tumor maximum diameter, or preoperative clinical stage (*P* > 0.05), as shown in Table 1. Comparing the clinical data and clinical stages of the training and validation sets, the results showed that there was no significant difference in age, sex, preoperative clinical stage, tumor maximum diameter, or pathological stage between the training set and the validation set (*P* > 0.05), as shown in Table 2.

**Table 1:** Comparison of clinical factors and clinical stages between SCLC and NSCLC patients (number).

**Clinical features**		**SCLC (*n* = 202)**	**NSCLC (*n* = 266)**	***p*-value**	***t*-value or χ^2^-value**
Sex	Male	152 (75.2%)	188 (70.7%)	0.272	1.207^*^
	Female	50 (24.8%)	78 (29.3%)		
Age (years)		61.6 ± 9.37	62.3 ± 9.62	0.401	0.840
Tumor maximum diameter (cm)		4.6 ± 2.5	4.9 ± 2.4	0.203	1.276
Smoking	Yes	160 (79.2%)	162 (60.9%)	<0.001	17.924^*^
	No	42 (20.8%)	104 (39.1%)		
Clinical stage	Early (I, II)	68 (33.7%)	101 (40.0%)	0.337	0.923^*^
	Late (III, IV)	134 (66.3%)	165 (60.0%)		

* *χ^2^-value (continuous variables were analyzed by the t-test and categorical variables were analyzed by the chi-square test)*.

**Table 2:** Composition ratio and clinical data of patients with different pathological types in the training and validation sets.

**Set**	**Number of cases**	**Age (years)**	**Sex**		**Smoking**	**Clinical staging**				**Tumor maximum diameter (cm)**	**Pathological type**			
			**M**	**F**		**I**	**II**	**III**	**IV**		**Small cell lung cancer**	**Squamous cell carcinoma**	**Adenocarcinoma**	**Large cell lung cancer**
Training set	327	62.2 ± 9.60	232	95	223	12	112	95	108	4.77 ± 2.42	141	70	70	46
Validation set	141	61.5 ± 9.33	108	33	97	6	45	46	44	4.80 ± 2.53	61	30	30	20
*t* or χ^2^		0.730	1.581^*^		0.016	0.766^*^				0.114			0.003^*^	
*p*-value		0.466	0.209		0.898	0.858				0.909			1	

* *χ^2^-value (continuous variables were analyzed by the t-test and categorical variables were analyzed by the chi-square test)*.

### The Predictive Efficacy of the Radiomics Signature for the Classification of SCLC and NSCLC

**Figure 3:**
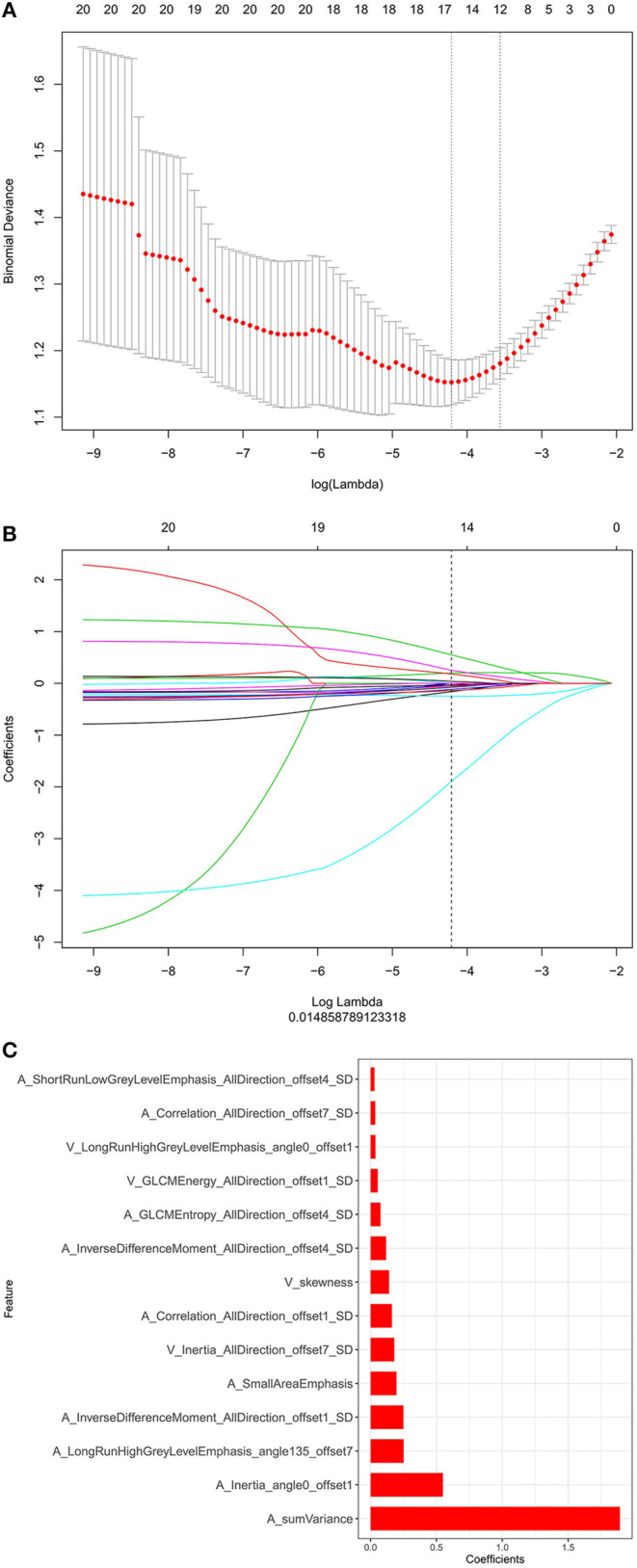
**(A)** The binomial deviation from the lasso regression cross-validation model is plotted as a log (λ) function by using the 10-fold cross-validation method. The y-axis represents binomial deviation, the lower x-axis represents log (λ), and the numbers above the x-axis represent the average number of predictive variables. The red dot represents the average deviation value of each model with a given λ, while the vertical bar of the red dot represents the upper and lower limit values of the deviation. The vertical dotted line represents the log (λ) value corresponding to the best λ value; the selection standard is the minimum standard. By adjusting different parameters (λ), the binomial deviation of the model is minimized, and the feature datasets with the best performance are selected. **(B)** Plots the coefficients of the log (λ) function. The λ value is the smallest at the dotted line. Select the coefficient that is not 0 here as the coefficient of the last reserved feature. **(C)** The y-axis shows the 14 feature names with non-zero coefficients retained at the minimum value of λ, and the x-axis shows their total coefficients in the lasso Cox analysis. The larger the coefficients are, the greater the predictive significance.

**Figure 4:**
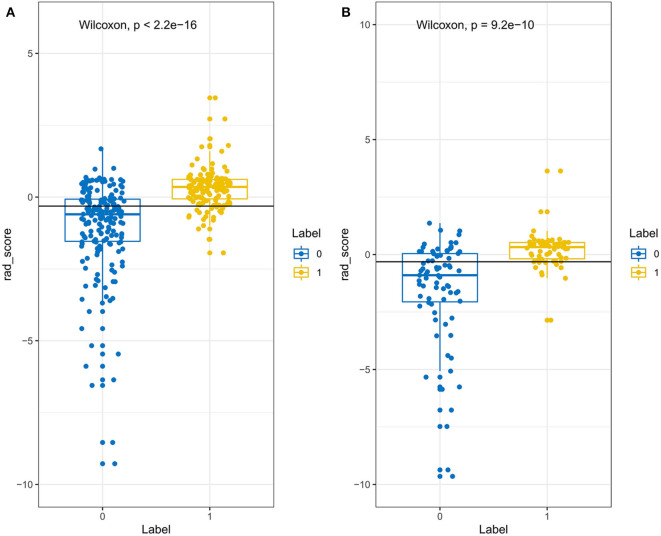
The Rad-score of each patient in the training set **(A)** and validation set **(B)**. The Rad-score is classified according to the threshold value. The Wilcoxon test was used to assess the difference between the two sets.

Through the reproducibility evaluation (inter- and intra datasets with a consistency coefficient >0.75) and the removal of highly correlated features (correlation coefficient >0.6), 14 features were screened out using lasso logistic regression, as shown in Figures 3A–C. Figure 4 shows the Rad-scores for each patient in the training and validation sets.

### Predictive Efficacy of the Radiomics Signature and the Radiomics Nomogram

**Figure 5:**
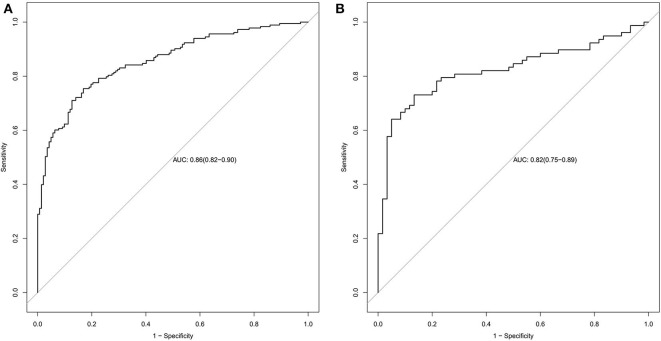
Radiomics signature ROC curves used to assess predictive performance. **(A)** The AUC of the training set is 0.86. **(B)** The AUC of the validation set is 0.82.

The radiomics signature established in this study has good ability to distinguish and predict the pathological types of SCLC and NSCLC. The AUC of the prediction model in the training set was 0.86 (95% CI: 0.82–0.90), and the AUC in the validation set was 0.82 (95% CI: 0.75–0.89), as shown in Figures 5A,B.

**Figure 6:**
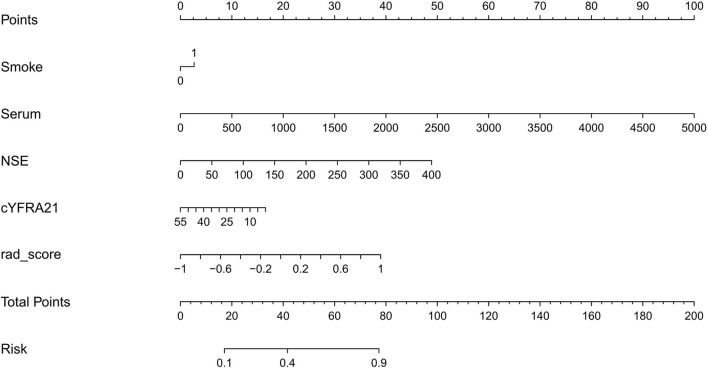
Radiomics nomogram for predicting SCLC and NSCLC.

Clinical factors found to be significantly associated with the classification of SCLC and NSCLC by univariate analysis are presented in Table 3. They include smoking and serum NSE and cYFRA21-1 values (*P* < 0.05 each). A clinical model was built based on the results of the multivariate logistic regression analysis of clinical variables. The results of multivariate logistic regression analysis suggested that smoking, serum NSE and cYFRA21-1 and Rad-score were independent predictors for the classification of SCLC and NSCLC (Table 4), with AUCs of 0.86 and 0.82, respectively. A radiomics nomogram incorporating the predictors, including smoking, NSE, cYFRA21-1 and Rad-score, was constructed (Figure 6).

**Table 3:** Positive results of univariate analysis for the classification of SCLC and NSCLC.

**Variables**	**OR (95% CI)**	***P*-value**
Smoking	2.35 (1.42–3.97)	<0.01
Serum	1.00 (1.00–1.00)	<0.01
NSE	1.03 (1.02–1.04)	<0.01
cYFRA21	0.93 (0.88–0.98)	0.01

**Table 4:** Positive results of multivariate logistic regression analysis for the classification of SCLC and NSCLC.

**Variables**	**OR (95% CI)**	***P*-value**
(Intercept)	0.42 (0.21–0.82)	0.01
Smoking	1.14 (0.57–2.28)	0.71
Serum	1.00 (1.00–1.00)	<0.01
NSE	1.01 (1.00–1.02)	0.07
cYFRA21	0.97 (0.91–1.02)	0.35
Rad-score	4.00 (2.55–6.70)	<0.01

**Figure 7:**
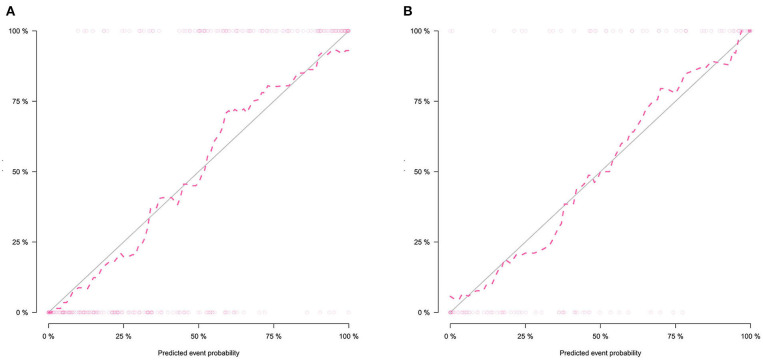
Calibration curves of the radiomics nomogram in the training set **(A)** and validation set **(B)**. The calibration curves show the calibration of the nomogram in terms of agreement between the predicted probability of SCLC and pathological findings. The 45° blue line indicates perfect prediction, and the dotted lines indicate the predictive performance of the nomogram. The closer the dotted line fit to the ideal line, the better the predictive accuracy of the nomogram.

**Figure 8:**
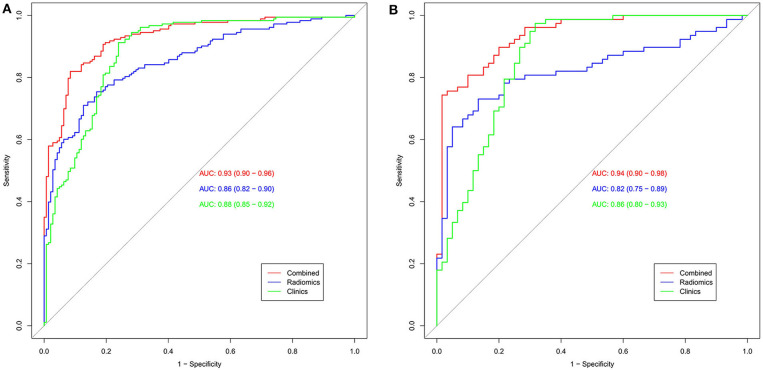
The AUC was used to estimate the predictive power of different models (**A**: training set; **B**: validation set). The radiomics signature and clinical model can be used for the classification of SCLC and NSCLC. In the validation set, the predictive ability of the nomogram (red, AUC = 0.94) was better than that of the clinical model (green, AUC = 0.86). The addition of clinical features improves the prediction efficiency of the radiomics signature.

**Figure 9:**
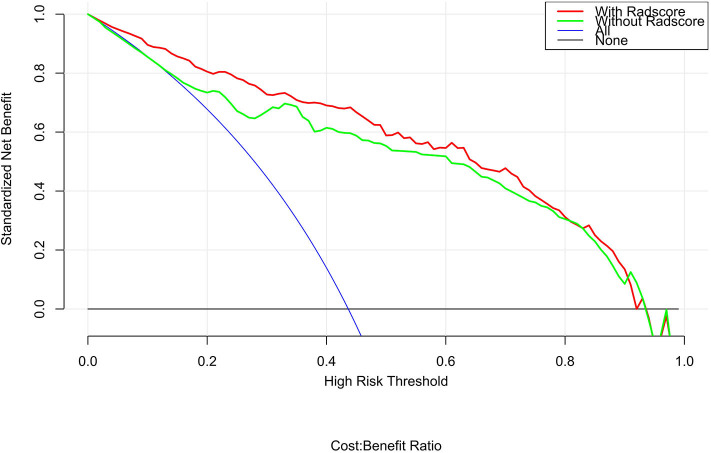
DCA for the radiomics nomogram. The y-axis shows the net benefit. The red line represents the radiomics nomogram. The blue line indicates the hypothesis that all patients had small cell lung cancer. The black line represents the hypothesis that no patients had small cell lung cancer. The x-axis shows the threshold probability, which is where the expected benefit of treatment is equal to the expected benefit of not undergoing treatment. The decision curves indicate that when the threshold probability is between 0.1 and 1, using the radiomics nomogram to predict small cell lung cancer adds more benefit than treating either all or no patients.

The calibration curve shows good agreement between the predicted probability of the nomogram and the actual probability (Figure 7). Compared with the results of the radiomics signature and clinical model, the nomogram has better prediction efficiency (Table 5 and Figure 8). In the training and validation sets, the AUC values were 0.93 (95% CI: 0.90–0.96) and 0.94 (95% CI: 0.90–0.98), and the accuracy was 0.85 (95% CI: 0.80–0.88) and 0.86 (95% CI: 0.79–0.92), respectively. The DCA for the radiomics nomogram is displayed in Figure 9, which shows that the radiomics nomogram is superior to the clinical model regarding the “treat all” vs. “treat none” strategies when the threshold probability is within the 0.1–1.0 range.

**Table 5:** Predictive ability of the radiomics nomogram, radiomics signature, and clinical model for the classification of SCLC and NSCLC.

**Variables**		**AUC**	**(95% CI)**	**Accuracy**	**Sensitivity**	**Specificity**
Clinical model	Train	0.88	(0.85–0.92)	0.84	0.84	0.84
	Test	0.86	(0.80–0.93)	0.84	0.83	0.85
Radiomics signature	Train	0.86	(0.82–0.90)	0.75	0.65	0.87
	Test	0.82	(0.75–0.89)	0.76	0.67	0.88
Radiomics nomogram	Train	0.93	(0.90–0.96)	0.85	0.80	0.88
	Test	0.94	(0.90–0.98)	0.86	0.85	0.87

## Discussion

In traditional single-energy CT imaging, tumors are assessed based on attenuation, morphology, and invasiveness. The effect of treatment is assessed based on changes in solid tumor volume and density (31). However, it is usually not possible to determine the pathological type of tumors based only on tumor morphology. Radiomics focuses on extracting a large number of quantitative imaging features, which can provide a detailed and comprehensive characterization of the tumor phenotype, and uses statistics and/or machine learning methods to screen the most valuable radiomics characteristics to analyze clinical information for the diagnosis and treatment of tumors (32–34). In recent years, a large number of basic studies have suggested that radiomics could evaluate tumor tissue characteristics in a non-invasive manner with high predictive accuracy (35, 36).

In this study, we observed 14 radiomics features with a significant association with the histological subtypes of lung cancer. The radiomics model established in this study has good predictive performance for the pathological classification of SCLC and NSCLC. The AUCs of the radiomics signature predictive model in the training set and the validation set were 0.86 and 0.82, respectively.

Furthermore, we found that clinical features including smoking status, NSE and cYFRA21 had potential ability to differentiate between SCLC and NSCLC. We built a radiomics nomogram including smoking status, NSE, cYFRA21, and Rad-score for individualized SCLC and NSCLC prediction. The AUC value of the radiomics nomogram in the validation set was 0.94, indicating that it has better predictive performance than the clinical model (AUC = 0.86) and the radiomics signature (AUC = 0.82). The accuracy, specificity and sensitivity were also improved, and the results of the validation set were as follows: accuracy: 86.2%; sensitivity: 84.7%; and specificity: 87.3%. The nomogram visualized the radiomic signature and clinical prediction factors into an easy-to-use tool for the individualized prediction of SCLC and NSCLC. In addition, calibration curves were constructed to indicate the performance of the radiomics nomogram for the classification of SCLC and NSCLC. The curves demonstrated good agreement between the predicted and observed values in the training and validation sets. In this study, central small cell lung cancer accounted for 67.3% of all small cell lung cancer cases, and in the non-small cell lung cancer group, the proportion of central NSCLCs was 60.5%. There was no significant difference between the two groups (*p* = 0.13). The previous reports (37) showed that central small-cell lung cancer accounted for ~90–95% of all small-cell lung cancer cases. In this study, central small-cell lung cancer accounted for a relatively low proportion. The possible reason is that some of the cases included in this study were surgical cases, while most small-cell lung cancers cannot be surgically removed, so the location results of lung cancer in this study may not be representative of the general population. Thus, this study did not introduce location as a feature of the study.

In 2002, Kido et al. (38) analyzed 70 cases of bronchial carcinoma (61 cases of adenocarcinoma and 9 cases of squamous cell carcinoma) by the fractal method. The results showed that the three-dimensional classification obtained from grayscale images was helpful in distinguishing adenocarcinoma from squamous cell carcinoma. Wu et al. (23) analyzed the relationship between radiomics features and the subtypes (adenocarcinoma and squamous cell carcinoma) of lung cancer. A total of 440 features were extracted in the study. After multivariate analysis and feature selection, the five most relevant features were applied, and the diagnostic efficiency (AUC) of the model was 0.72. Junior et al. (25) found that the AUCs of the training group and the validation group were 0.71 and 0.81, respectively, when the radiomics features of lung cancer CT images were used to distinguish adenocarcinoma, squamous cell carcinoma and large cell carcinoma, which indicated that the radiomics method had great potential in the diagnosis of the histopathological subtypes of lung cancer. One study in 2018 (26) showed that the radiomics signature established by lasso logistic regression model can distinguish adenocarcinoma and squamous carcinoma well. The AUCs of the training set and validation set were 0.905 and 0.893, respectively. Linning et al. (27, 28) found that the use of a radiomics approach for classifying the histological subtypes of lung cancer demonstrated potential for differentiating AD and SCC, as well as AD and SCLC; however, the approach showed relatively low performance in classifying SCC and SCLC. For classifying AD and SCC, AD and SCLC, and SCC and SCLC, the AUCs were 0.801, 0.857, and 0.657 (non-enhanced); 0.834, 0.855, and 0.619 (arterial phase); and 0.864, 0.864, and 0.664 (venous phase), respectively. According to their studies (27, 28), the prediction efficiency of the model based on enhanced CT was better than or equal to that based on non-contrast CT imaging, and non-contrast CT was not available in many cases due to the lack of thin-layer recombination images in our study. As a result, non-contrast CT was not used to extract CT radiomics features, and only dual-phase enhanced CT was independently analyzed to establish predictive models in our study. The AUCs of our model in the training and validation sets were 0.93 and 0.94, respectively, which were higher than the previous results. One of the possible reasons may be that our study included a larger sample size, and the other may be that we added clinically relevant prediction parameters, which may make our results more comprehensive and accurate. In our study, we included samples of all major lung cancer subtypes, including SCLC, adenocarcinoma, squamous cell carcinoma, and large cell lung cancer. Our findings suggest that some robust radiomics features have great potential for the classification of SCLC and NSCLC. The established radiomics nomogram has a better prediction ability for the classification of SCLC and NSCLC, which require different treatment options. We believe that our work may serve as a promising diagnostic tool for the classification of SCLC and NSCLC in a non-invasive manner, allowing clinicians to select the appropriate treatment plan for lung cancer patients.

This study has certain limitations. First, this study used only contrast-enhanced CT image features and did not compare the classification performance with models established by positron emission tomography (PET) imaging or other imaging modalities such as non-contrast CT. These all need further study. Second, this study is a retrospective study, and there may be bias in case selection. Extracting texture features from artificially segmented data makes it difficult to remove small blood vessels and bronchi in nodules or masses, which may affect the accuracy of certain features. Third, this study is a single-center retrospective study. Although this study used a cross-validation method and the amount of data was repeatedly calculated and verified, the number of cases in this study was relatively small and could not meet the requirements of a large number of samples, which may lead to instability. In the future, we will try to increase the sample size and carry out multicenter joint research.

In conclusion, the radiomics signature we established has good performance for the classification of SCLC and NSCLC, and we also developed and validated the first nomogram with better diagnostic performance for the classification of SCLC and NSCLC based on the radiomics signature and clinical factors.

## Data Availability Statement

All datasets generated for this study are included in the article/supplementary material.

## Ethics Statement

The studies involving human participants were reviewed and approved by the Institutional Review Board of the Affiliated Hospital of Qingdao University.

## Author Contributions

QF, ShiL, and ShuL conceived the project, analyzed the data, and wrote the paper. HY, YH, and XT participated in data collection and processing. QF, CZ, WX, and XL provided expert guidance and reviewed the manuscript. All authors edited the manuscript. Thanks to Professor Wenhong Wang, Jigang Wang, and Zhimin Wei for their guidance and help in pathology related work.

## Conflict of Interest

The authors declare that the research was conducted in the absence of any commercial or financial relationships that could be construed as a potential conflict of interest.
